# Risk of Edentulism Among Older Adults with Multimorbidity

**DOI:** 10.3390/dj14050295

**Published:** 2026-05-13

**Authors:** Rolla Mira, Wael Sabbah

**Affiliations:** Faculty of Dentistry, Oral & Craniofacial Sciences, King’s College London, London WC2R 2LS, UK; rolla.mira@kcl.ac.uk

**Keywords:** ageing, longitudinal studies, multimorbidity, tooth loss

## Abstract

**Objective:** This study aimed to assess whether older American adults with multimorbidity are at higher risk of becoming edentate over time. **Methods:** We used data from three waves of the Health and Retirement Study (HRS), a longitudinal survey of older American adults aged 50 years and over. Data on multimorbidity was from 2012, while data on complete tooth loss was from 2018. Multimorbidity included five common and serious conditions, namely diabetes, heart conditions, lung diseases, cancer, and stroke. Socioeconomic factor was indicated by total wealth in 2006; behaviour was indicated by smoking in 2012. We used Structural Equation Modelling (SEM) to assess the relationship between multimorbidity in 2012 and complete tooth loss in 2018. Participants with complete tooth loss in 2012 were excluded from the analysis. **Results:** Among 6286 participants with complete data across all three waves, each additional chronic condition in 2012 was associated with 1.30 times higher odds of edentulism in 2018 (95% CI: 1.12, 1.52). In the SEM, multimorbidity in 2012 was positively associated with being edentate in 2018 (estimate: 0.01, 95% CI 0.01, 0.02); smoking and wealth were also significantly associated with edentulism. Wealth and smoking were also associated with multimorbidity. **Conclusions:** Older adults with multimorbidity appear to have a higher probability for becoming edentate. The findings highlight the need for oral health promotion activities for those with multimorbidity.

## 1. Introduction

Complete tooth loss, or edentulism, remains a significant health burden affecting millions of older adults worldwide [1,2]. In the United States, approximately 17% of adults aged 65 years and older are edentate, representing a substantial decline in oral health status and quality of life [3]. The consequences of edentulism extend beyond oral health, affecting nutrition, speech, psychological well-being, overall health and mortality [4,5]. Edentate older adults experience reduced nutritional intake, compromised dietary diversity, and increased susceptibility to nutritional deficiencies that can complicate the management of chronic diseases [1].

Concurrently, multimorbidity—defined as the simultaneous presence of two or more chronic diseases—has emerged as a dominant healthcare challenge in the ageing populations [6]. Estimates suggest that multimorbidity affects more than one-third of older adults, with prevalence increasing substantially with advancing age. Common chronic conditions in older adults include diabetes, coronary heart disease, stroke, chronic respiratory diseases, and cancer. These conditions frequently coexist and interact in complex ways that amplify healthcare burden and complicate clinical management [7,8]. The economic implications of multimorbidity are substantial, with individuals having multiple chronic conditions accounting for disproportionate healthcare expenditures.

The relationship between systemic health conditions and oral health outcomes has been documented in cross-sectional studies [9,10,11]. Diabetes Mellitus is a known systemic condition that affects whole-body systems including periodontal tissues leading to loss of bone structure and tooth loss. Several studies have demonstrated its association with accelerated periodontal disease progression and tooth loss through multiple biological pathways [12,13]. Cardiovascular diseases have been linked to poor oral health through both direct inflammatory pathways and indirect mechanisms related to functional limitations and healthcare access inequalities. The bidirectional relationship between periodontitis and cardiovascular disease has been well established, with evidence suggesting that periodontal infection contributes to systemic inflammation and atherosclerosis progression [14,15]. Older adults struggling with the management of multimorbidity could also have a shift in priority, leading to neglect of oral self-care and the deterioration of oral hygiene and subsequent oral diseases, including dental caries, periodontitis and tooth loss [16]. Furthermore, the management of multimorbidity often requires being on multiple medications which could lead to xerostomia, which reduce saliva’s protective function in terms of lubrication and neutralization, leading to increases in caries and periodontal diseases, and subsequently tooth loss [17,18]. That aside, there are common risk factors that could lead to both chronic conditions and poor oral health; these include adverse socioeconomic conditions, health risk behaviours such as smoking and excessive alcohol consumption, and stresses [19,20].

However, few longitudinal investigations have prospectively examined whether the accumulation of chronic conditions (multimorbidity) represents an independent risk factor for complete tooth loss in ageing populations [21]. On the other hand, the reversed association between complete tooth loss and multimorbidity has also been observed [22].

Given the growing prevalence of both edentulism and multimorbidity, understanding their temporal relationship is essential for developing targeted prevention and intervention strategies. Previous research from Brazil demonstrated that multimorbidity was associated with tooth loss in a nationally representative survey [23], yet evidence from large-scale American longitudinal studies remains limited.

This study addresses this gap by utilizing national longitudinal data from the United States. The objective of this study is to assess whether older American adults with multimorbidity are at higher risk of becoming edentate over a six-year follow-up period. We hypothesize that the cumulative burden of multiple chronic conditions would be associated with increased edentulism risk, independent of age, gender, socioeconomic factors and smoking behaviour.

## 2. Methods

### 2.1. Study Design and Population

This analysis utilized data from three waves of the Health and Retirement Study (HRS), a nationally representative longitudinal survey of American adults aged 50 years and older. The HRS employs a complex multi-stage stratified cluster sampling design and includes biennial in-person and telephone interviews. Additional data on nutrition and biomarkers are also collected for subsamples. The study began in 1992 and continues to collect comprehensive data on health, economic, and social outcomes. Data from the biennial files were included in this study from three time points: 2006 (baseline for socioeconomic factors), 2012 (assessment of multimorbidity and smoking status), and 2018 (assessment of complete tooth loss). All data used in this analysis are available in the public biennial data files of the HRS.

Older American individuals selected from the HRS, a nationwide sample of Americans 50 years of age and older, make up the study sample. The majority of longitudinal surveys of older persons begin recruiting participants at age 50 in order to track changes in their social and health conditions as they age [24].

The analytic sample included participants who had complete data available across all three waves and were not edentate in 2012.

### 2.2. Measures

Multimorbidity in 2012 was assessed based on self-reported diagnosis of five common chronic conditions: diabetes, coronary heart disease, lung disease, cancer, and stroke. These conditions were selected based on their high prevalence in the American population, and clinical significance among older adults [25]. We acknowledge that there are other chronic conditions, but they were perceived to be less serious and less common. Furthermore, earlier studies using the same database used the same set of chronic conditions [22]. A multimorbidity count score was calculated ranging from zero to five conditions, reflecting the cumulative burden of chronic disease. This approach to measuring multimorbidity burden has been validated in previous epidemiological studies and captures the additive effects of multiple chronic conditions [26,27]. The variable for multimorbidity ranged from 0 to 5 and was used as the main exposure in the analysis.

Complete Tooth Loss: In 2018, participants were asked about the status of their natural teeth. Those reporting no natural teeth remaining were classified as edentate. Participants who reported complete tooth loss in 2012 were excluded from the analysis to enable the assessment of incident edentulism and establish temporality between the exposure and the outcome.

Socioeconomic Status: Total wealth (including home and financial assets) was measured in 2006 and categorized into quartiles. Wealth quartiles were utilized as an indicator of socioeconomic position over the longitudinal period, as wealth is considered a more stable measure of socioeconomic status than income in older populations.

Smoking status was recorded in 2012. Participants who reported they have not smoked more than a hundred cigarettes in their lifetime were considered non-smokers. Smoking was classified into three categories: never smoked, former smoker, and current smoker. Smoking represents a well-established risk factor for periodontal disease and tooth loss and was included as a key behavioural variable.

Demographic factors included age (in years) and gender (male/female) and were recorded at baseline.

### 2.3. Statistical Analysis

Participants who were lost to follow-up over the survey years and who did not have complete data for all included variables were excluded from the analysis. Descriptive statistics were calculated to characterize the study population by tooth loss status. Chi-square tests were employed to assess associations between categorical variables and edentulism status. Analysis of variance was utilized to compare mean age and multimorbidity count between dentate and edentate groups.

Structural Equation Modelling (SEM) was used [28] to simultaneously model relationships between multimorbidity, socioeconomic factors (wealth), behavioural factors (smoking), and edentulism while accounting for demographic covariates. The SEM approach allowed for the examination of both direct pathways and potential indirect relationships between variables. This methodology is particularly suited for longitudinal data analysis as it can simultaneously model multiple relationships and account for complex patterns of association. The conceptual model depicts that socioeconomic factors at baseline will impact behaviours and multimorbidity over time, and they impact edentulism directly and indirectly through behaviours and multimorbidity. Subsequentially, multimorbidity and behaviours directly impact edentulism. All variables were used in their original format (no latent variables). The maximum likelihood with missing values (MLMV) estimation method was used in CFA and SEM analysis. The Tucker–Lewis Index (TLI), the comparative fit index (CFI), and the root mean square error of approximation (RMSEA) were used to assess the goodness of fit. A satisfactory model fit is indicated by an RMSEA value < 0.05, a CFI and TLI > 0.90 [29]. Standardized coefficients, 95% confidence interval (CI), and *p*-values are used to show the results.

## 3. Results

**Figure 1:**
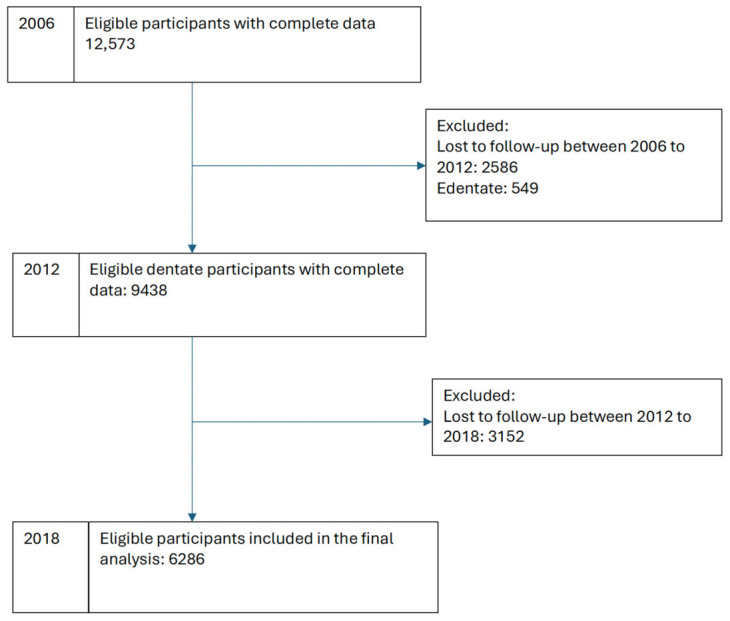
Flowchart of the number of participants included in the analysis from 2006 to 2018.

The analysis included 6286 participants with complete data in all waves and were dentate in 2012 (Figure 1). Table 1 shows the demographic and clinical characteristics of the study population and their association with complete tooth loss. The mean age of participants in 2006 was 62.6 years (95% CI: 62.4, 62.8) with 62% being female. The mean multimorbidity count in 2012 was 0.58 (95% CI: 0.56, 0.60).

In 2018, 295 participants (4.69%) had developed complete tooth loss. Males demonstrated a higher incidence of edentulism (6.03%) compared to females (3.87%). The mean of multimorbidity in 2012 was significantly higher among edentate (0.72) in 2018 than dentate participants (0.57). There were clear wealth gradients in edentulism. Smoking in 2012 was also significantly associated with edentulism in 2018 (Table 1). Complete tooth loss was much higher among men than women, but there was no significant difference in mean age between being dentate and edentate.

In a univariate analysis, the odds for complete tooth loss in 2018 was 1.31 (95% Confidence Interval “CI”: 1.3, 1.53), indicating a 30% increase in the risk of complete tooth loss for each additional chronic condition.

Table 2 exhibits the results of the path analysis from the SEM. The analysis revealed that multimorbidity in 2012 maintained a significant positive association with edentulism in 2018 (path estimate: 0.01, 95% CI: 0.01, 0.02) accounting for age, gender, wealth, and smoking status.

The path analysis further demonstrated that lower wealth was associated with both multimorbidity and edentulism. Smoking status showed similar patterns, with current smoking associated with increased multimorbidity burden and elevated edentulism (Table 2).

**Figure 2:**
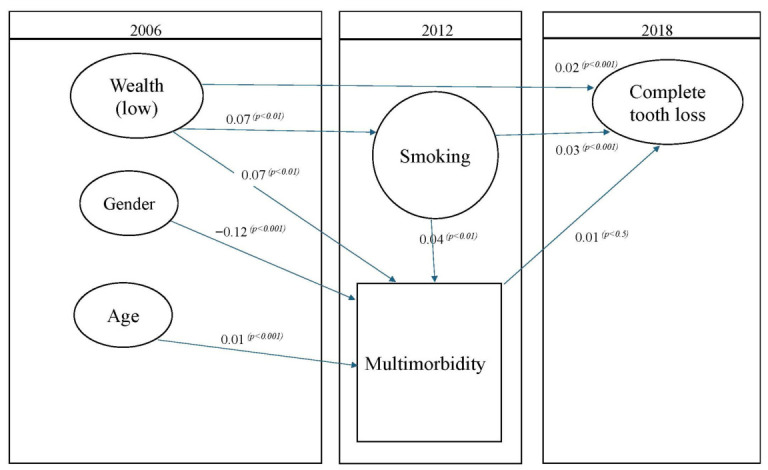
Structural Equation Modelling (SEM) of edentulism among older adults with multimorbidity.

Figure 2 shows the pathway between each of wealth, multimorbidity and edentulism, and smoking, multimorbidity and edentulism. The diagram in Figure 2 demonstrates that wealth at baseline was directly and significantly associated with a greater risk of edentulism in 2018, and indirectly though behaviours and multimorbidity. Furthermore, each of multimorbidity and smoking in 2012 were directly and significantly associated with edentulism in 2018.

## 4. Discussion

This longitudinal investigation provides novel evidence that older American adults with multimorbidity have a higher probability of complete tooth loss over time. The finding that each additional chronic condition confers a 30% higher in odds of edentulism underscores the cumulative impact of chronic disease burden on oral health outcomes. The SEM findings further substantiate this association by demonstrating a persistent independent relationship between multimorbidity and edentulism after adjustment for important confounders. Given the observational nature of the study, the observed associations reflect temporality, but not causality.

The findings of this analysis are consistent with other studies in China and Brazil which found that individuals with multimorbidity are at higher risk of tooth loss [21,30]. Both studies used functional dentition as the dependent outcome but did not demonstrate temporality. The study from China assessed the association between having two or more conditions and functional dentition among older adults, while the Brazilian study examined adults between age 18 and 59 and included 13 medical conditions. The current study has the advantage of demonstrating temporal relationship between five common chronic conditions and edentulism. The association was also observed in a cross-sectional survey of American adults [16].

Several mechanisms could explain the observed association between multimorbidity and edentulism. First, chronic diseases such as diabetes and cardiovascular disease directly compromise periodontal health through inflammatory mechanisms, accelerating bone resorption and tooth loss. Diabetes, in particular, impairs immune function and enhances inflammatory responses that are central to periodontal disease pathogenesis. Hyperglycemia promotes advanced glycation end products (AGEs) that contribute to chronic inflammation and impair wound healing in periodontal tissues [31]. Earlier studies demonstrated biological pathways between systemic conditions and tooth loss [19,20]. Second, individuals with multimorbidity often experience functional limitations, reduced mobility, and polypharmacy that may impair oral hygiene maintenance and access to preventive dental care [17]. Polypharmacy can also cause xerostomia (dry mouth), which increases susceptibility to caries and periodontal disease [18]. Third, the psychological burden of managing multiple chronic conditions may reduce adherence to oral health behaviours and engagement with dental services. Individuals with complex healthcare needs may prioritize the management of systemic diseases over preventive dental care. Fourth, biological pathways linking systemic inflammation to periodontal disease may be particularly pronounced in individuals with multiple chronic conditions. Multimorbidity is characterized by systemic inflammation, which amplifies the pathogenic cascade leading to periodontal tissue destruction [19,20]. Furthermore, participants suffering from multiple chronic conditions, particularly when they are serious and life-threatening, could neglect oral health-promoting behaviours, and could also lose the ability for self-care. Furthermore, excessive medical burdens undoubtedly impact the use of dental services due to conflict with medical appointments. The cost of medications and any medical appointments for the treatment of multimorbidity add financial constraints, with patients more likely to forfeit dental care.

Common risk factors for both chronic conditions and tooth loss, and the social determinants of health could also contribute to the observed association, with chronic conditions preceding tooth loss. Unsurprisingly, in this study, lower wealth was associated with each of multimorbidity and tooth loss. The strong socioeconomic gradient in edentulism and its association with multimorbidity aligns with the established literature [32], demonstrating that lower socioeconomic status predicts both greater chronic disease burden and worse oral health outcomes. Individuals with limited financial resources face substantial barriers to accessing preventive and restorative dental care, placing them at elevated risk for tooth loss. Additionally, socioeconomic inequalities are often accompanied by clustering of health risk factors, including smoking, suboptimal nutrition, and reduced healthcare engagement, which collectively increase both multimorbidity and edentulism risk [33]. This is particularly relevant given that earlier analysis of the same database demonstrated a reversed association between tooth loss and multimorbidity [22].

The finding that smoking emerged as an independent risk factor for edentulism in this analysis is consistent with extensive prior literature. Smoking accelerates periodontal disease through multiple pathways including impaired immune response, reduced periodontal blood flow, and direct toxic effects on oral tissues [34]. The association between smoking and multimorbidity burden further suggests that tobacco use represents a critical intervention target for reducing both systemic disease burden and oral health complications.

### Clinical and Public Health Implications

Both clinical practice and public health policy will be significantly impacted by these findings. First, dentists should prioritize screening and care for their older patients with multimorbidity and chronic disease. This is because these patients need more frequent professional supervision and more intensive preventative measures. The early identification of high-risk people may be facilitated by the implementation of systematic screening for major chronic illnesses in dental settings.

Second, the need for integrated, multidisciplinary approaches to oral health promotion that involve patients across healthcare settings where they receive treatment for chronic illnesses is also highlighted by the strong correlation between edentulism and multimorbidity. In addition to being encouraged to counsel patients regarding dental care engagement, physicians and other primary care providers should have a greater understanding of the reciprocal relationship between oral health and systemic disease. Interprofessional cooperation between dental and medical professionals may improve the detection and treatment of edentulism risk factors.

Third, smoking cessation programmes are a highly effective way to lower multimorbidity and edentulism in older adults. To increase the incentive for quitting, public health campaigns and therapeutic interventions should particularly highlight the negative effects of tobacco smoking on oral health. Chronically ill older persons should have access to community-based smoking cessation services.

Fourth, addressing socioeconomic determinants of health represents a fundamental public health priority. The expansion of dental insurance coverage for low-income older adults, the elimination of cost barriers to preventive dental care, and the development of community-based dental services accessible to vulnerable populations deserve serious policy attention.

This study has some limitations worth highlighting. The study’s observational nature precludes conclusive causal inference. The observed relationships may be explained by unmeasured confounding variables, even though longitudinal data enables us to demonstrate temporal precedence. Remaining confounding by elements like oral hygiene habits or dental care use patterns cannot be ruled out. A second possibility is measuring error in self-reported oral health status. However, prior research has tested the self-reporting of edentulism against clinical evaluation and shown that it has acceptable validity. There was a considerable number of excluded participants with no data in all waves, but this is inevitable in longitudinal surveys, particularly among older adults. Finally, even though we included five major chronic conditions in the multimorbidity measurements, it did not fully account for the full disease load that certain participants would encounter. Although we acknowledge that the multimorbidity assessment might have been strengthened by including other disorders like depression, arthritis, or cognitive impairment, there is a very large number of chronic conditions that would compromise the analysis if they were all included.

## 5. Conclusions

This analysis of longitudinal data from the HRS provides evidence that older persons in the United States who have multiple chronic conditions are at significantly higher risk of losing all of their teeth. Even after controlling for behaviours and socioeconomic position, the cumulative burden of chronic illness still seems to be a risk factor for edentulism. These results highlight the necessity of initiating oral health promotion strategies that target those with multiple chronic conditions. The underlying causes of the observed association, the significance of dental care access and utilization patterns, and the efficacy of therapies aimed at lowering the incidence of edentulism in this susceptible group should all be investigated in future studies. There is great potential for improving oral health outcomes for older persons with multiple comorbidities by using preventative methods at the junction of medical and dental treatment.

## Figures and Tables

**Table 1:** Participant characteristics and baseline demographics. Data from the Health and Retirement Study (HRS) in 2006, 2012, and 2018. (N = 6286).

Characteristic		Percentages/Mean	Percentage Edentate in 2018	*p*-Value *
Gender	Male	37.9%	6.0%	<0.001
	Female	62.1%	3.9%	
Wealth quartiles “2006”	Highest	36.8%	2.2%	<0.001
	2nd Highest	29.6%	4.4%	
	2nd Lowest	22.1%	6.8%	
	Lowest	11.4%	9.5%	
Smoking Status “2012”	Never smoked	49.2%	3.2%	<0.001
	Former smoker	42.4%	4.7%	
	Current smoker	8.4%	12.9%	
Mean age (95% CI)	Dentate	62.6 (62.4, 62.8)	62.5 (62.4, 62.8)	0.763
	Edentate		62.8 (61.7, 63.8)	
Mean of multimorbidity “2012” (95% CI)	Dentate	0.58 (0.56, 0.60)	0.57 (0.55, 0.59)	<0.001
	Edentate		0.72 (0.63, 0.81)	

* *p*-value from Chi-Square and *t*-test.

**Table 2:** Structural Equation Modelling path analysis results for the association between multimorbidity and edentulism among older American adults (HRS) for 2006–2018 (N = 6286).

Pathway	Estimate	95% CI	*p*-Value
Edentulism (2018)			
Multimorbidity (2012)	0.01	(0.01, 0.02)	<0.01
Lower Wealth (2006)	0.02	(0.01, 0.03)	<0.001
Smoking (2012)	0.03	(0.02, 0.04)	<0.001
Multimorbidity (2012)			
Lower Wealth (2006)	0.07	(0.05, 0.08)	<0.001
Smoking (2012)	0.04	(0.02, 0.07)	<0.01
Age	0.01	(0.01, 0.02)	<0.001
Gender	−0.12	(−0.15, −0.08)	<0.001
Smoking (2012)			
Lower Wealth (2006)	0.07	(0.05, 0.08)	<0.001
Model fit			
RMSEA	0.028	(0.021, 0.041)	
CFI	1.00		
TLI	1.00		

Path estimates represent standardized coefficients from Structural Equation Modelling. All pathways were estimated simultaneously in the SEM model.

## Data Availability

Data is publicly available on https://hrs.isr.umich.edu/about (accessed on 30 January 2026).
